# STIM1 and STIM2-mediated Ca^2+^ influx regulates antitumour immunity by CD8^+^ T cells

**DOI:** 10.1002/emmm.201302989

**Published:** 2013-08-06

**Authors:** Carl Weidinger, Patrick J Shaw, Stefan Feske

**Affiliations:** Department of Pathology and Cancer Institute, New York University School of MedicineNew York, NY, USA

**Keywords:** CTL, SOCE, STIM1, STIM2, tumour immunity

## Abstract

Store-operated calcium entry (SOCE) through Ca^2+^ release-activated Ca^2+^ (CRAC) channels regulates the function of many immune cells. Patients with loss-of-function mutations in the CRAC channel genes *ORAI1* or *STIM1* are immunodeficient and are prone to develop virus-associated tumours. This and the reported role of Ca^2+^ signals in cytotoxic lymphocyte function suggest that SOCE may be critical for tumour immune surveillance. Using conditional knock out mice lacking STIM1 and its homologue STIM2, we find that SOCE in CD8^+^ T cells is required to prevent the engraftment of melanoma and colon carcinoma cells and to control tumour growth. SOCE is essential for the cytotoxic function of CTLs both *in vivo* and *in vitro* by regulating the degranulation of CTLs, their expression of Fas ligand and production of TNF-α and IFN-γ. Our results emphasize an important role of SOCE in antitumour immunity, which is significant given recent reports arguing in favour of CRAC channel inhibition for cancer therapy.

→See accompanying article http://dx.doi.org/10.1002/emmm.201303129

## INTRODUCTION

Modulation of intracellular Ca^2+^ concentrations [Ca^2+^]_i_ is an important signal transduction mechanism to regulate the function of immune cells (Feske, 2007; Vig & Kinet, 2009). In T cells, antigen binding by the T-cell receptor (TCR) results in a [Ca^2+^]_i_ rise that is due to Ca^2+^ release from the endoplasmic reticulum (ER) and Ca^2+^ influx across the plasma membrane through Ca^2+^ release-activated Ca^2+^ (CRAC) channels. Opening of CRAC channels is required for sustained [Ca^2+^]_i_ increases, activation of Ca^2+^ dependent signalling molecules and T-cell effector functions (Feske, 2007). The pore of the CRAC channel is formed by ORAI1 proteins in the plasma membrane. Channel opening and Ca^2+^ influx results from the binding of two proteins, stromal interaction molecules (STIM) 1 and STIM2, to ORAI1. STIM proteins are located in the membrane of the ER, where they sense [Ca^2+^]_ER_ using an EF hand Ca^2+^ binding domain in their N terminus. Ca^2+^ release from the ER causes oligomerization of STIM molecules, their translocation to the plasma membrane and binding to ORAI1. This process is called store-operated Ca^2+^ entry (SOCE) and constitutes the predominant Ca^2+^ influx pathway in lymphocytes (Feske, 2007).

Both STIM1 and STIM2 are required for SOCE in T cells. Loss-of-function or null mutations in the human *STIM1* gene abolish Ca^2+^ influx in T cells and cause immunodeficiency in affected patients (Byun et al, 2010; Fuchs et al, 2012; Picard et al, 2009). Similarly, deletion of murine *Stim1* severely impairs SOCE and the function of T cells (Oh-Hora et al, 2008), most evident in the inability of STIM1-deficient CD4^+^ T cells to mediate inflammation in animal models of autoimmune disease (Ma et al, 2010; McCarl et al, 2010; Schuhmann et al, 2010). The role of SOCE in CD8^+^ T cell-mediated immunity *in vivo* is less well defined. SOCE-deficient patients with mutations in *STIM1* or *ORAI1* genes are susceptible to recurrent viral infections, potentially due to impaired CD8^+^ T-cell function and elimination of virus-infected cells (Feske, [Bibr b9],[Bibr b10]). In several cases, chronic viral infections in ORAI1- and STIM1-deficient patients have caused Epstein–Barr virus (EBV)-positive B cell lymphoma and Human herpesvirus (HHV) 8-associated Kaposi sarcoma (Byun et al, 2010; Fuchs et al, 2012), suggesting that SOCE may be required for antitumour immunity by CD8^+^ T cells.

CD8^+^ T cells are cytotoxic lymphocytes (CTLs) that play an important role in antitumour immune responses because of their ability to kill tumour cells (Frey & Monu, 2008; Schwarz et al, 2012). Infiltration of tumours with CD8^+^ T cells positively correlates with survival, for instance in patients with small cell lung cancer (Kawai et al, 2008). CTLs recognize tumour cells through their T-cell antigen receptor and CD8 coreceptor which bind to (tumour) peptide-MHC class I complexes on the surface of tumour cells. TCR engagement activates several CTL effector functions that contribute to antitumour immunity (Chavez-Galan et al, 2009). One is the release of perforin and granzyme from cytolytic granules, which induce caspase-dependent apoptotic cell death in their target cells (Voskoboinik et al, 2010). Early evidence suggested that CTL effector functions depend on Ca^2+^ as human CTLs exhibited Ca^2+^ influx upon immune synapse formation with tumour cells (Lyubchenko et al, 2001). Furthermore, chelating extracellular Ca^2+^ with EGTA impaired the ability of murine CTLs to kill lymphoma cells (MacLennan et al, 1980). A possible explanation is the dependence of CTLs on Ca^2+^ influx to form immune synapses and to release cytolytic granules (Pores-Fernando & Zweifach, 2009). However, genetic evidence for a role of CRAC channels in the cytolytic function of CTLs is missing. In natural killer (NK) cells, degranulation and cytotoxicity depend on SOCE as the lytic function of NK cells from an ORAI1-deficient patient was strongly reduced. By contrast, the cytolytic function of CD8^+^ T cells from a STIM1-deficient patient was normal despite strongly reduced SOCE (Fuchs et al, 2012). Other mechanisms contribute to the antitumour immune function of CD8^+^ T cells including their ability to express of death receptor ligands FasL and TRAIL (Chavez-Galan et al, 2009; Mahmood & Shukla, 2009) and to secrete IFN-γ and TNF-α (Calzascia et al, 2007). Importantly however, the roles of SOCE in these CD8^+^ T-cell effector functions and especially in tumour immunosurveillance *in vivo* are not understood.

To determine if CRAC channels control CD8^+^ T-cell functions in the context of antitumour immunity, we used mice with T cell-specific deletion of *Stim1* and *Stim2* genes (*Stim1*^*fl/fl*^*Stim2*^*fl/fl*^ *Cd4Cre*, or DKO for simplicity) that lack SOCE in CD4^+^ and CD8^+^ T cells (Oh-Hora et al, 2008). We found that SOCE in T cells curtails the growth of tumour allografts and that STIM1 and STIM2 deficient CTLs fail to prevent tumour cell engraftment. SOCE was not required for the expansion of tumour specific CTLs and their infiltration of tumours, but was necessary for lytic granule exocytosis, tumour cell killing and production of IFN-γ and TNF-α. Collectively, our data support an important role of SOCE in antitumour immune responses.

## RESULTS

### SOCE in CD8^+^ T cells curtails tumour growth *in vivo*

**Figure 1:**
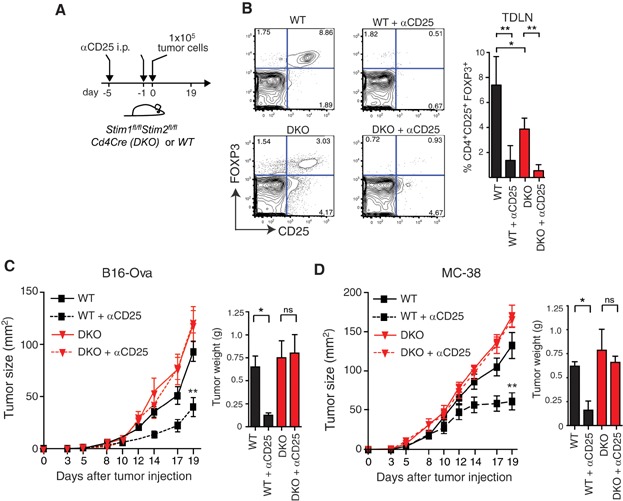
SOCE in CD8^+^ T cells is required to curtail tumour growth *in vivo* **A.** Allogenic tumour transfer to Treg cell-depleted mice. Wildtype (WT) and *Stim1*^*fl/fl*^*Stim2*^*fl/fl*^ *Cd4Cre* (DKO) mice were injected i.p. with 250 µg anti-CD25 antibody (PC61; or 1× PBS as control) on Days −5 and −1 before intradermal (i.d.) tumour inocculation with 1 × 10^5^ B16-Ova melanoma cells or MC-38 adenocarcinoma cells.**B.** Efficiency of Treg depletion. Three weeks after anti-CD25 injection, lymphocytes were isolated from tumour draining lymph nodes (TDLN) of WT and DKO mice and analysed for CD4^+^ CD25^+^ FOXP3^+^ Treg cells by FACS. *n* = 4 mice per group, *p < 0.05,**p < 0.001.**C,D.** Tumour growth in WT and DKO mice. WT (black) and DKO (red) mice injected with B16-Ova melanoma (C) and MC-38 adenocarcinoma (D) were monitored for tumour size (left panels); after mice were sacrificed, tumour weight was measured on day 19 (bar graphs). Dashed lines represent mice injected with anti-CD25 to deplete Treg cells. *n* = 7 (C) and *n* = 6 (D) mice per group. *p < 0.05, **p < 0.01.

To test whether CTL-mediated tumour immunosurveillance depends on SOCE, we used several tumour allograft models by injecting *Stim1*^*fl/fl*^*Stim2*^*fl/fl*^ *Cd4Cre* (DKO) mice with syngeneic cancer cells. Note that in these mice STIM1 and STIM2 protein expression is deleted in both CD4^+^ and CD8^+^ T cells, which show a complete lack of SOCE (Oh-Hora et al, 2008). When we initially injected DKO mice intradermally with 1 × 10^5^ B16-Ova melanoma cells, we did not observe significant differences in tumour growth compared to wildtype (WT) control mice (data not shown and Fig 1C). Tumour growth in both WT and DKO mice was rapid, presumably due to the known low immunogenicity of B16-Ova cells. To enhance antitumour immune responses by CTLs and to inhibit the accumulation of immunosuppressive Treg cells in B16 tumours, we depleted CD4^+^ CD25^+^ FOXP3^+^ regulatory T (Treg) cells (Fig 1A), which have been shown to increase antitumour CD8^+^ T-cell responses in mice and in human patients, resulting in attenuated murine tumour growth (Ataera et al, 2011; Rech et al, 2012). Intraperitoneal injection of WT and DKO mice with anti-CD25 antibodies resulted in significant depletion of FOXP3^+^ Treg cells in lymphoid organs 3 weeks after antibody treatment (Fig 1B). To determine if SOCE is required for CTL-mediated antitumour immunity, we compared the growth of B16-Ova melanoma and MC-38 colon carcinoma cells in DKO and WT littermate control mice following Treg cell depletion. In WT mice, the growth of B16 melanomas and MC-38 carcinomas was significantly reduced compared to untreated WT mice (Fig 1C and D). By contrast, DKO mice failed to efficiently control tumour growth despite depletion of Treg cells. Similar observations were made without prior depletion of Treg cells when WT and DKO mice were injected with more immunogenic EG7 lymphoma cells. Under these conditions, WT mice successfully rejected EG7 tumour cells despite initial tumour growth, whereas tumours continuously grew in *Stim1*^*fl/fl*^*Stim2*^*fl/fl*^ *Cd4Cre* mice (unpublished observations). Collectively, our data show that SOCE in CD8^+^ T cells mediated by STIM1 and STIM2 is critical for antitumour immunity.

### SOCE in CD8^+^ T cells is not required for the migration of CTLs

**Figure 2:**
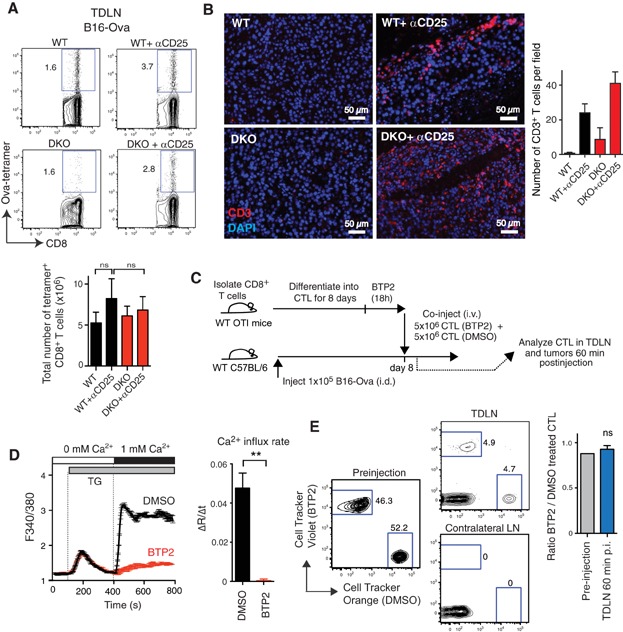
SOCE is not required for the priming of tumour specific CD8^+^ T cells or their infiltration of tumours Expansion of tumour-specific CD8^+^ T cells *in vivo*. Nineteen days after i.d. injection of Treg-depleted WT and *Stim1*^*fl/fl*^*Stim2*^*fl/fl*^ *Cd4Cre* (DKO) mice with B16-Ova melanoma cells, lymphocytes were isolated from TDLN and the frequencies and total numbers of Ova-specific CD8^+^ T cells were determined by staining with SIINFEKL (Ova) MHC-class I (H-2K^b^) tetramers. Representative FACS plots and mean ± SEM of *n* = 4 mice per group.T-cell infiltration of tumours. Nineteen days after injection of tumour cells into Treg-depleted WT and DKO mice, tumours were excised, paraffin-embedded and stained with anti-CD3 antibody (red) and DAPI (blue) to detect tumour-infiltrating T cells. Bar graphs show mean ± SEM of total numbers of CD3^+^ T cells per 20× field (three fields per tumour analysed).Migration of tumour antigen-specific cytotoxic CD8^+^ T cells (CTLs) into TDLN. (C) Experimental design. WT C57BL/6 mice were injected i.d. with 1 × 10^5^ B16-Ova melanoma cells. Eight days later, mice were injected i.v. with CD8^+^ T cells that had been isolated from WT OT-1 mice and differentiated into Ova-specific CTLs for 8 days *in vitro*. To inhibit SOCE, CTLs were treated with BTP2 (1 μM) or vehicle (DMSO) for 18 h before coinjection. 5 × 10^6^ DMSO-treated and 5 × 10^6^ BTP2-treated CTLs were labelled with CellTracker Orange (CMTMR) and Cell Tracker Violet, respectively.Calcium influx after treatment with BTP2. CTLs from WT OT-1 mice were generated as described in (C) and treated for 18 h with BTP2 or DMSO, labelled with Fura-2-AM in the absence of BTP2 (30 min) and rested for 20 min. SOCE was induced by CTL stimulation with 1 μM thapsigargin (TG) in Ca^2+^-free Ringer solution (0 mM Ca^2+^) followed by readdition of 2 mM Ca^2+^. Representative graphs and mean ± SEM of Ca^2+^ influx rates after readdition of Ca^2+^ from *n* = 3 independent experiments performed in duplicates (**p < 0.001).CTL infiltration of TDLN. Analysis of Ova-specific CD8^+^ T cells from OT-1 mice in TDLN and contralateral LN 60 min after coinjection of BTP2- and DMSO-treated CTLs. Representative FACS plots and mean ratio ± SEM of *n* = 3 mice per group.

The failure of STIM1/STIM2-deficient T cells to control tumour growth in DKO mice could be due to several mechanisms including impaired priming and expansion of tumour antigen-specific CD8^+^ T cells, lack of migration into tumours, abolished cytotoxic function or a combination of the above. We first analysed whether tumour antigen-specific CD8^+^ T cells are present in tumour draining lymph nodes (TDLNs) of DKO and WT mice injected with B16-Ova melanoma cells. The percentages and number of Ova-specific CD8^+^ T cells within TDLNs, assessed by staining with SIINFEKL (Ova) MHC-class I (H-2K^b^) tetramers, was comparable in WT and DKO mice (Fig 2A). We next tested if SOCE-deficient T cells can infiltrate tumours by analysing the number of T cells in WT and DKO mice. Following Treg cell depletion, the number of CD3^+^ T cells found within tumours increased dramatically, but no significant differences in cell numbers were apparent between WT and DKO mice (Fig 2B). To directly investigate if SOCE is required for the homing of tumour antigen-specific CTLs, we adoptively transferred Ova-specific CTLs into mice with established B16-Ova melanomas. Naïve CD8^+^ T cells were isolated from wildtype OT-1 mice (that express a TCR specific for the Ova peptide SIINFEKL and H-2K^b^) and differentiated into CTLs for 8 days *in vitro*. To inhibit SOCE, CTLs were incubated with the long-acting, selective CRAC channel inhibitor BTP2 for the last 18 h before adoptive transfer into tumour bearing mice (Fig 2C). Treatment of Ova-specific CTLs with 1 µM BTP2 resulted in almost complete inhibition of SOCE, which persisted for at least 1 h after BTP2 had been removed from cells (Fig 2D). To test if SOCE-deficient CTLs are able to home to TDLN, we coinjected CTLs treated with either BTP2 or DMSO (control) at a 1:1 ratio into mice with established B16-Ova melanomas. One hour after injection, no difference in the ratio of BTP2- and DMSO-treated CTLs was observed in TDLN (Fig 2E). BTP2-treated CTL homed to TDLN to the same extent as DMSO-treated CTL, indicating that SOCE is not required for CTL migration. Collectively, our data show that SOCE is not required for the priming and expansion of tumour antigen-specific CTLs or their migration into TDLN.

### SOCE in CTLs prevents engraftment of tumour cells

**Figure 3:**
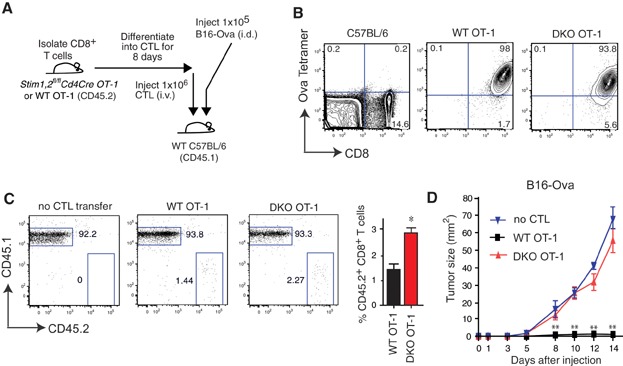
SOCE in CTLs prevents engraftment of tumour cells Adoptive transfer of Ova-specific CTLs. CD8^+^ T cells from WT OT-1 and *Stim1*^*fl/fl*^*Stim2*^*fl/fl*^ *Cd4Cre* OT-1 (DKO) mice were differentiated into CTLs for 8 days *in vitro*. 1 × 10^6^ Ova-specific CTLs were injected (i.v.) into WT CD45.1 mice on the day of intradermal (i.d.) injections of 1 × 10^5^ B16-Ova melanoma cells.Frequencies of Ova-specific CD8^+^ T cells before injection into CD45.2 mice. T cells are stained with anti-CD8 antibody and SIINFEKL (Ova) MHC-class I (H-2K^b^) tetramers. FACS plots are representative of 2 independent experiments.Frequencies of CD45.2^+^ WT OT-1 and DKO OT-1 CTLs in the blood of recipient mice (CD45.1) mice 5 days after CTL transfer. Representative FACS plots and mean ratio ± SEM of *n* = 4 (WT) and *n* = 5 (DKO) mice per group; *p < 0.05.Tumour growth in CTL-transferred mice. Tumour size was monitored in recipient mice inoculated i.d. with B16-Ova melanoma cells and that had received no CTLs (blue), WT OT-1 CTLs (black) or DKO OT-1 CTLs (red). Graphs are representative of *n* = 4 (WT), *n* = 5 (DKO) and *n* = 3 (no CTLs) mice per group. **p < 0.001.

To understand why DKO mice fail to control tumour growth (Fig 1) despite the generation of tumour specific CD8^+^ T cells and the ability of SOCE deficient CTLs to infiltrate tumours and home to TDLNs, we analysed whether SOCE-deficient CTLs can prevent tumour engraftment. For this purpose, we isolated CD8^+^ T cells from *Stim1*^*fl/fl*^*Stim2*^*fl/fl*^ *Cd4Cre OT-1* or wildtype OT-1 mice (CD45.2^+^) and differentiated them into Ova-specific CTLs *in vitro*. After 8 days, CTLs were coinjected together with B16-Ova melanoma cells into wildtype C57BL/6 (CD45.1^+^) mice (Fig 3A). In contrast to experiments described above, in this setting all differences observed between mice injected with WT and DKO CTLs are due to CD8^+^ T-cell intrinsic effects of SOCE. We confirmed that the number of Ova-specific T cells and TCR expression level are comparable between CTLs derived from WT and DKO OT-1 mice (Fig 3B). To exclude that lack of SOCE in CTLs from DKO OT-1 mice affected their survival *in vivo*, we determined the number of CD8^+^ CD45.2^+^ CTLs in the blood of recipient mice 5 days after adoptive transfer. In fact, the frequencies of STIM1/2-deficient Ova-specific CTLs were higher than those of wildtype CTLs (Fig 3C). Importantly, when we compared the growth of B16-Ova melanomas in mice that had received either no CTLs, wildtype CTLs or DKO CTLs, we observed a complete failure of STIM1/2-deficient CTLs to prevent tumour progression (Fig 3D). 14 days after melanoma and CTL injection, no tumours were detectable in mice that had received wildtype CTLs, whereas tumour growth was similar in mice that were injected with STIM1/2-deficient CTLs or no CTLs at all. This failure of STIM1/2-deficient CTLs to prevent tumour engraftment was not due to their reduced number since no significant differences of transferred CTLs could be found in the spleens (∼1.5 × 10^4^ WT vs. ∼1.5 × 10^4^ DKO-derived CTLs) and TDLN (∼2 × 10^3^ WT vs. ∼1.8 × 10^3^ DKO-derived CTLs) of mice. Taken together, our data show an important CD8^+^ T-cell intrinsic role for SOCE in the ability of CTLs to prevent tumour engraftment and growth.

### Cytolytic effector functions of tumour specific CTLs require SOCE

**Figure 4:**
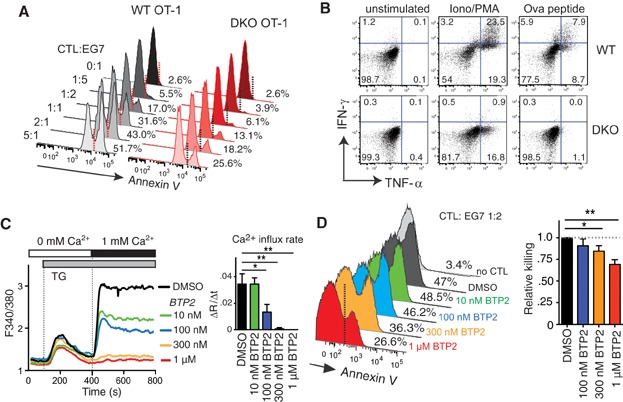
Cytotoxicity of tumour-specific CTLs depends on SOCE Impaired cytolytic function of STIM1/STIM2-deficient CTLs. Ova specific CD8^+^ T cells from WT OT-1 and *Stim1*^*fl/fl*^*Stim2*^*fl/fl*^ *Cd4Cre* OT-1 (DKO) mice were differentiated into CTLs for 8 days *in vitro*, co-cultured with CellTracker Orange (CMTMR)-labelled EG7-Ova lymphoma cells at the indicated CTL:EG7 ratios. After 90 min, cells were stained with Annexin V and analyzed for apoptotic cell death by flow cytometry. Percentages indicate Annexin V^+^ CMTMR^+^ EG7-Ova tumour cells.Cytokine production. WT and DKO CTLs were left untreated or stimulated with either 20 nM PMA/1 µM Ionomycin or 25 ng/ml Ova-peptide. After 6h, cells were stained with antibodies against CD8, TNF-α and IFN-γ. Cytokine expression was determined by flow cytometry.Inhibition of SOCE by BTP2. CD8^+^ T cells from WT OT-1 mice were differentiated into CTLs for 8 days *in vitro*. On Day 8, cells were treated with the indicated concentrations of the CRAC channel inhibitor BTP2 for 14 h, labelled with Fura-2-AM in the absence of BTP2 and rested for 20 min. CTLs were stimulated with 1 µM thapsigargin (TG) and SOCE was measured after readdition of 1 mM Ca^2+^. Representative graphs and mean Ca^2+^ influx rates (after readdition of Ca^2+^) ± SEM of *n* = 3 independent experiments performed in duplicates. *p < 0.05, **p < 0.001.Cytolytic function of CTLs. CTLs treated with different concentrations of BTP2 (or DMSO as control) for 14 h were coincubated with CMTMR-labelled EG7-Ova lymphoma cells at a 1:2 ratio. After 90 min, cells were labelled with Annexin V and analyzed by flow cytometry. Representative dot plots and the mean ± SEM of four to six independent experiments performed in duplicates are shown. Relative cytotoxicity was calculated for each experiment as (% CMTMR^+^ Annexin V^+^ cells coincubated with BTP2-treated CTLs)/(% CMTMR^+^ Annexin V^+^ cells coincubated with DMSO-treated CTLs). *p < 0.05; **p < 0.001.

Since the expansion and tumour infiltration of CTLs is independent of STIM1 and STIM2, we speculated that cytolytic effector functions of tumour-specific CD8^+^ T cells may require SOCE. To test this hypothesis, we differentiated Ova-specific CD8^+^ T cells from wildtype OT-1 and *Stim1*^*fl/fl*^*Stim2*^*fl/fl*^ *Cd4Cre OT-1* mice into CTLs *in vitro* and cocultured them with EG7-Ova cells that are derived from the murine T-cell lymphoma EL4 (Moore et al, 1988). STIM1/2-deficient Ova-specific CTL showed a 29–64% reduction in their cytotoxic function compared to wildtype CTL depending on the CTL:EG7 ratio (Fig 4A). In addition, CTLs from *Stim1*^*fl/fl*^*Stim2*^*fl/fl*^ *Cd4Cre OT-1* mice had a severe defect in the production of IFN-γ and TNF-α compared to wildtype CTLs when stimulated with either PMA/ionomycin or Ova peptide *in vitro* (Fig 4B). To confirm these findings and analyse the quantitative relationship between SOCE and CTL function, we inhibited CRAC channel function with increasing concentrations of BTP2. 10 nM and 100 nM BTP2 partially inhibited SOCE in CTLs, whereas 300 nM and 1 µM BTP2 almost completely abolished SOCE (Fig 4C) similar to CTLs from *Stim1*^*fl/fl*^*Stim2*^*fl/fl*^ *Cd4Cre* mice (Oh-Hora et al, 2008). To determine the effects of dose-dependent inhibition of SOCE on the cytolytic function of CTLs on tumour cells, we cocultured EG7-Ova cells with CTLs pretreated with increasing concentrations of BTP2. At the highest concentration of BTP2 (1 µM), cytolytic killing of EG7-Ova cells was inhibited ∼31% compared to CTLs not treated with BTP2 and thus normal SOCE (Fig 4D).

**Figure 5:**
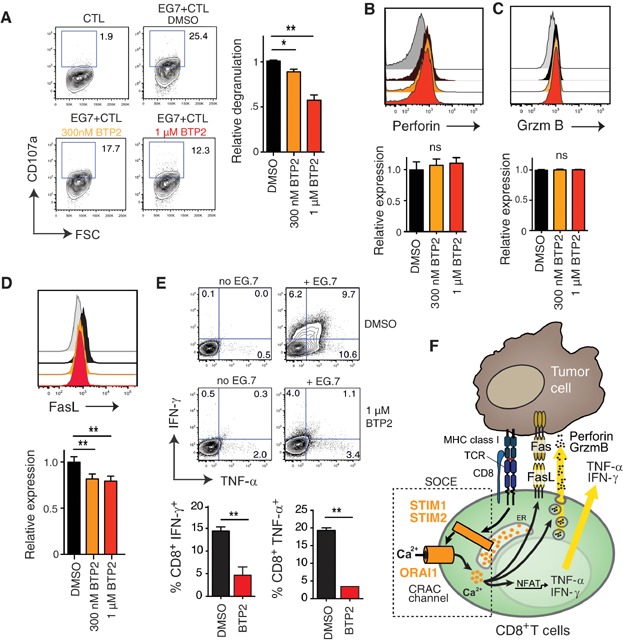
Several cytolytic functions of CTLs require SOCE **A.** CTL degranulation. Ova specific CTLs from WT OT-1 mice were treated with BTP2 for 14 h and analysed for CD107a (LAMP-1) expression by flow cytometry after coincubation for 90 min with EG7-Ova cells at a 1:2 ratio (same experiment as in Fig 4D). Representative dot plots and means ± SEM of *n* = 3 independent experiments performed in duplicates. Relative degranulation for each experiment was calculated as: (% CD8^+^ CD107a^+^ of BTP2 treated cells/% CD8^+^ CD107a^+^ of DMSO treated cells). *p < 0.01, **p < 0.001.**B,C.** Expression of perforin and granzyme B. CTLs were treated with 300 nM (orange) or 1 µM (red) BTP2 or DMSO (black) for 14 h and cocultured with EG7-Ova lymphoma cells for 90 min at a 1:2 ratio. Expression of perforin (panel B) and granzyme B (panel C) was determined by flow cytometry using specific antibodies. Grey histograms show DMSO-treated CTLs not incubated with EG7-Ova cells. Representative histograms (top) and means ± SEM (bottom) of two experiments performed in duplicates are shown. Relative perforin and granzyme B expression was calculated as: (MFI BTP2-treated CTL)/(MFI DMSO-treated CTL). ns, not significant.**D.** Fas ligand expression. CTLs were treated with 300 nM or 1 μM BTP2 for 14 h, washed and cocultured with EG7-Ova lymphoma cells for 90 min at a 1:2 ratio. Expression of FasL was determined by flow cytometry. Representative histograms and means ± SEM of *n* = 2 experiments performed in duplicates are shown. Relative FasL expression was calculated as: (MFI BTP2-treated cells)/(MFI DMSO-treated cells). Grey histogram depicts unstimulated CTLs. **p < 0.01.**E.** IFN-γ and TNF-α production. CTLs were treated with 1 µM BTP2 (or DMSO) for 14 h and cocultured with EG7-Ova cells at a 1:2 ratio for 6 h in the presence of Brefeldin A. IFN-γ and TNF-α production were analyzed by flow cytometry. Representative dot plots and means ± SEM of *n* = 2 independent experiments performed in duplicates. **p < 0.01.**F.** SOCE in CTL function. TCR stimulation results in the activation of STIM1 and STIM2, opening of ORAI1-CRAC channels and SOCE. The subsequent intracellular Ca^2+^ increase mediates IFN-γ and TNF-α production, FasL expression and degranulation of CTLs, which are required for antitumour immunity by CD8^+^ T cells.

CTLs utilize a number of mechanisms to kill tumour cells including release of cytolytic granules containing perforin and granzyme B, as well as the expression of the death receptor ligand FasL that interact with the Fas receptors on target cells, respectively. Inhibition of CRAC channel function with BTP2 significantly inhibited the exocytosis of lytic granules by Ova-specific CTLs in response to coincubation with EG7-Ova cells as measured by cell surface expression of CD107a (Fig 5A). By contrast, the expression of proteins contained in lytic granules, Granzyme B and Perforin, was not dependent on SOCE (Fig 5B and C) at least in response to TCR stimulation of Ova-specific CTLs following coincubation with EG7-Ova tumour cells. A moderate defect in granzyme B protein expression was observed in CTLs from *Stim1*^*fl/fl*^*Stim2*^*fl/fl*^ *Cd4Cre OT-1* mice (that lack all SOCE) following strong stimulation with PMA/ionomycin or Ova peptide (unpublished observations). Inhibition of SOCE with BTP2 also resulted in a moderate but significant decrease in the expression of FasL on the surface of OT-1 CTLs after coculture with EG7-Ova cells (Fig 5D). This is consistent with reports that the transcription of FasL is regulated by NFAT, a Ca^2+^ dependent transcription factor (Rengarajan et al, 2000; Wang et al, 2011). Apart from contact dependent cytotoxicity, CTL control tumour growth by secretion of TNF-α and IFN-γ (Calzascia et al, 2007). Inhibition of SOCE in OT-1 CTLs with BTP2 severely impaired the production of both cytokines after coculture with EG7-Ova cells (Fig 5E). Collectively, our data show that CRAC channel function and SOCE mediate important effector functions of cytotoxic T cells that are required for killing of tumour cells.

## DISCUSSION

Taken together, our results show for the first time that activation of CRAC channels by STIM1 and STIM2 proteins is essential for tumour immunosurveillance by CD8^+^ T cells. STIM1 and STIM2 are dispensable for the priming, expansion and homing of tumour antigen-specific CTLs. However, they are crucial for cytolytic effector functions of CTLs, especially their ability to produce IFN-γ and TNF-α, to release perforin-containing cytolytic granules, to induce FasL and to kill tumour cells. Our data confirm previous observations of decreased degranulation and cytotoxicity by CTLs after chelating extracellular Ca^2+^ with EGTA (MacLennan et al, 1980). However, the channels mediating the Ca^2+^ influx required for the cytolytic function of tumour specific CTLs were not well defined. Our data using conditional knockout mice with T-cell specific deletion of *Stim1* and *Stim2* genes unambiguously show that the Ca^2+^ influx required for antitumour immunity by CTLs is dependent on CRAC channels, which are activated by STIM1 and STIM2. As a consequence, CTL lacking both *Stim1* and *Stim2* genes and therefore CRAC channel function failed to control the growth of melanoma and colon carcinoma cells and to prevent tumour engraftment. The role of SOCE in T-cell mediated antitumour immunity is summarized in Fig 5F.

Impaired antitumour immunity and CTL function in *Stim1*^*fl/fl*^*Stim2*^*fl/fl*^ *Cd4Cre* mice likely explain why human patients lacking SOCE due to mutations in the CRAC channel genes *ORAI1* and *STIM1* develop virus-associated malignancies such as EBV^+^ B cell lymphoma and HHV8^+^ Kaposi sarcoma (Byun et al, 2010; Fuchs et al, 2012). Initially, T cells require SOCE to control viral infections because *Stim1*^*fl/fl*^*Stim2*^*fl/fl*^ *Cd4Cre* mice showed impaired effector and memory CD8^+^ T cells responses and subsequently developed chronic viral infections (unpublished observations). In addition, the data we present here show that once malignant transformation has occurred, SOCE in CD8^+^ T cells is required for antitumour immunity and the cytolytic function of CD8^+^ T cells.

It is noteworthy that the cytolytic function of CTLs was only impaired when SOCE was completely abolished in CD8^+^ T cells from *Stim1*^*fl/fl*^*Stim2*^*fl/fl*^ *Cd4Cre* mice or after inhibition of WT CTLs with high doses of the CRAC channel inhibitor BTP2. Cytolytic functions of CTLs *in vitro* were largely intact in CD8^+^ T cells from *Stim1*^*fl/fl*^ *Cd4Cre* and *Orai1*^*R93W*^ knock-in mice (data not shown), which have strongly impaired but not abolished SOCE (McCarl et al, 2010; Oh-Hora et al, 2008). Consistent with these findings, we observed normal rejection of tumour cells in Treg-depleted *Stim1*^*fl/fl*^ *Cd4Cre* mice *in vivo* compared to WT littermates (unpublished observations). STIM1 and STIM2 have synergistic roles in CRAC channel activation and both molecules are required for full SOCE in CD8^+^ T cells. STIM1 and STIM2 act with different kinetics and thresholds of activation in response to store depletion (Shaw et al, 2012). Only the combined deletion of *Stim1* and *Stim2* genes completely abolishes SOCE. Deletion of *Stim1* alone strongly, but incompletely reduces SOCE. By contrast, deletion of *Stim2* alone has no effect on the peak of Ca^2+^ influx but significantly impairs sustained SOCE (Oh-Hora et al, 2008). Normal tumour rejection in *Stim1*^*fl/fl*^ *Cd4Cre* mice therefore indicates that moderate SOCE is sufficient for the cytolytic effector functions of CTLs and their ability to provide antitumour immunity *in vivo*. This might also explain why CD8^+^ T cells from a patient with homozygous loss-of-function mutation in *STIM1* and severely impaired SOCE showed normal cytotoxicity against CD3-coated L1210 target cells *in vitro* (Fuchs et al, 2012). This is in contrast to impaired cytolytic function of human NK cells lacking functional ORAI1, the pore-forming subunit of the CRAC channel, which had a severe defect in killing L1210 and K562 tumour cells (Maul-Pavicic et al, 2011). Similarly, STIM1-deficient murine NK cells showed defective cytotoxicity *in vitro* (Maul-Pavicic et al, 2011), pointing to a potential difference in the SOCE requirements of CTLs and NK cells for their cytolytic function. Collectively, these data from STIM1, STIM1/STIM2 and ORAI1 deficient patients and mice demonstrate that SOCE is required for the cytotoxicity of CTLs, but that moderate levels of SOCE are sufficient for CTL function.

Recent studies have demonstrated an important role for ORAI1 and STIM1 proteins in cancer cells and carcinogenesis. For instance, SOCE was shown to control the proliferation and apoptosis of cancer cells, promote tumour metastasis and regulate neovascularization [reviewed in (Bergmeier et al, 2013)]. Dysregulated expression of *ORAI1* and *STIM1* genes was found in several types of cancer and has been linked to more aggressive forms of breast and cervical cancer (Chen et al, 2011; Yang et al, 2009). CRAC channel inhibition has therefore been proposed as a potential treatment for certain forms of cancer. Our findings indicate that drugs targeting CRAC channels and inhibiting SOCE will compromise antitumour immunity mediated by CD8^+^ T cells and, most likely, other immune cells such as NK cells and dendritic cells. Therefore, a careful analysis of the effects of global CRAC channel inhibition on immune responses against different types of cancer is necessary to avoid unwanted effects on cancer progression.

**The paper explained**

PROBLEM:

Recognition and elimination of tumour cells by the immune system is an important mechanism to prevent the development and spread of tumours. Cytotoxic CD8^+^ T cells play an important role in antitumour immunity by recognizing and killing malignant cells expressing tumour antigens. Calcium (Ca^2+^) signals were reported to contribute to the cytotoxic function of CD8^+^ T cells, but which Ca^2+^ channels and Ca^2+^ dependent effector functions mediate antitumour immunity is poorly understood.

RESULTS:

In this study, we investigated the role of Ca^2+^ release-activated Ca^2+^ (CRAC) channels and the Ca^2+^ influx they mediate, called store-operated calcium entry (SOCE), in antitumour immunity. CRAC channels are composed of the ORAI1 pore protein and two activating molecules, stromal interaction molecule (STIM) 1 and 2. SOCE mediated by ORAI1, STIM1 and STIM2 is the main Ca^2+^ influx pathway in T lymphocytes. We used mice with T cell-specific deletion of *Stim1* and *Stim2* genes, which have severely reduced SOCE (*Stim1*-deficient mice) or completely abolished SOCE (*Stim1/Stim2*-deficient mice). We found that STIM1 and STIM2 in T cells are required to curtail tumour growth in animal models of melanoma and adenocarcinoma. SOCE in cytotoxic CD8^+^ T cells was essential to control the engraftment of tumour cells as only wild-type CD8^+^ T cells but not those lacking STIM1 and STIM2 prevented tumour growth. SOCE was not required for the generation and expansion of tumour specific CD8^+^ T cells or their migration to tumours. However, the exocytosis of cytolytic granules by CD8^+^ T cells, their expression of FasL at the cell surface and the production of IFN-γ and TNF-α was dependent on SOCE. Accordingly, CD8^+^ T cells lacking STIM1 and STIM2 failed to kill tumour cells both *in vitro* and *in vivo*.

IMPACT:

Our data show that STIM1 and STIM2 are key regulators of cytotoxic T-cell function *in vitro* and *in vivo*. We show for the first time that SOCE mediated by STIM1 and STIM2 is required for antitumour immune responses. In light of recent findings that SOCE promotes carcinogenesis and proposals to use inhibitors of SOCE for anticancer therapy, our findings demonstrate that drugs inhibiting the Ca^2+^ channels that mediate SOCE would compromise antitumour immunity and promote tumour growth.

## MATERIALS AND METHODS

### Mice

*Stim1*^*fl/fl*^ *Cd4Cre* and *Stim1*^*fl/fl*^*Stim2*^*fl/fl*^ *Cd4Cre* (DKO) mice were described previously (Oh-Hora et al, 2008). They were further crossed to OT-1 mice (stock # 003831) expressing a transgenic TCR specific for the SIINFEKL peptide of ovalbumin (Ova). OT-1 and CD45.1 (stock # 002014) mice were purchased from Jackson Laboratories (Bar Harbor, ME). Mice were maintained in a specific pathogen free barrier facility at New York University Langone Medical Center and used in accordance with protocols approved by the institutional animal care and use committee.

### Tumour allografts and Treg depletion

B16-Ova melanoma cells, MC-38 colon carcinoma cells and EG7-Ova T-cell lymphoma cells were gifts from Drs. J. Allison (Memorial Sloan Kettering Cancer Institute, NY), A. Frey (NYU Medical Center, NY) and A. Erlebacher (NYU Medical Center, NY), respectively. Tumour cells were grown in RPMI 1640 medium supplemented with 10% foetal calf serum. 1 × 10^5^ B16-Ova and MC-38 tumour cells were injected intradermally (i.d.) into the flank of mice. Tumour size was assessed *in situ* using a caliper; tumour weight was measured after tumour excision. For Treg depletion, mice were injected with 0.25 mg anti-CD25 antibody (clone PC61, BioXcell, West Lebanon, NH) or 1× PBS (control) on Days 5 and 1 before tumour cell transfer.

### Flow cytometry, antibodies and tetramers

Antibodies against the following proteins were purchased from eBiosciences (San Diego, CA): CD4 (GK1.5), CD8 (53–6.7), CD25 (eBio3C, PC61), CD107a (1D4B), FOXP3 (FJK-16s), TNF-α (MP6-XT22), IFN-γ (XMG1.2), Perforin (eBioOMAK-D), Granzyme B (NGZB) and FasL (MFL3). Annexin V–FITC and Annexin V–eFluor 450 were from BD Bioscience (San Jose, CA). H-2Kb Ova SIINFEKL tetramers were generated by the MHC Tetramer Production Facility at Baylor College Medicine (Houston, TX). Intracellular cytokine staining was performed as described (McCarl et al, 2010). Flow cytometry was conducted using a LSR II (BD Biosciences) and analysed using FlowJo software (TreeStar, Ashland, OR).

### Immunohistochemistry

Tumours were excised, fixed in 4% formaldehyde overnight and embedded in paraffin. 5 μm sections were used for immunostaining as described (Nancy et al, 2012). Briefly, tissues were incubated with biotinylated anti-CD3 antibody (2C11, 1:500, eBioscience) followed by horseradish peroxidase (HRP)-conjugated streptavidin (Jackson Immunoresearch, West Grove, PA). The antibody signal was amplified with biotin-tyramide (Perkin Elmer) and incubation with Alexa Fluor 594-conjugated Streptavidin (Invitrogen). Cell nuclei were counterstained with DAPI (Invitrogen). Epifluoresence images were acquired on an AxioImager M1 microscope and analyzed using AxioVision software (both Carl Zeiss Microimaging, Thornwood, NY). Images were uniformly processed to enhance signal intensity using ImageJ software (http://rsb.info.nih.gov/ij/).

### Intracellular Ca^2+^ measurements

CD8^+^ T cells were labelled with 2 µM Fura-2-AM (Invitrogen, Carlsbad, CA) for 30 min at 22–25°C, washed and kept in RPMI 1640 medium until use. Measurements of intracellular [Ca^2+^]_i_ were conducted using a FlexStation 3 microplate reader (Molecular Devices, Sunnyvale, CA). Baseline [Ca^2+^]_i_ was acquired in 0 mM Ca^2+^ Ringer solution containing (in mM) 155 NaCl, 4.5 KCl, 3 MgCl_2_, 10 d-glucose, 5 Na-HEPES. After 120 s, cells were stimulated with 1 µM of the Sarco/Endoplasmic reticulum ATPase (SERCA) inhibitor thapsigargin (EMD Millipore, Billerica, MA). After 420 s, Ca^2+^-containing Ringer solution (2 mM CaCl_2_) was added to the cells (final [Ca^2+^]_o_ 1 mM). Cells were excited at 340 and 380 nm and fluorescence emission measured at 510 nm every 5 s. The Ca^2+^ influx rate (Δ*R*/Δ*t*) was calculated 20 sec (*t*_20_) after readdition of Ca^2+^ (*t*_0_) using the equation (*R*[*t*_20_] − *R*[*t*_0_])/(*t*_20_ − *t*_0_), with *R* being the Fura-2 emission ratio 340/380 nm. Graphs were plotted using Graphpad Prism software. For some experiments, cells were pretreated for 14–18 h with the CRAC channel inhibitor BTP2 (*N*-(4-[3,5-bis(trifluoromethyl)-1*H*-pyrazol-1-yl]phenyl)-4-methyl-1,2,3-thiadiazole-5-carboxamide (EMD Millipore, Billerica, MA) at the indicated concentrations.

### Adoptive CTL transfer

Ova-specific CTLs were generated by isolating splenocytes from the spleens of wildtype OT-1 mice and by stimulation of CD8^+^ T cells with 0.75 µg/ml SIINFEKL peptide. CTLs were differentiated for 6–8 days as previously described (Budhu et al, 2010; McCarl et al, 2010). Starting on Day 3, RPMI 1640 medium was supplemented with 100 U/ml human rIL-2 (NIH AIDS reagent program). On Day 8, CD8^+^ CTLs were purified using a negative selection kit (Stemcell Technologies, Vancouver, Canada). For homing assays, CTLs were labelled with either 5 µM CellTracker Orange or CellTracker Violet (Invitrogen, Carlsbad, CA). 1 × 10^6^ CTLs were injected i.v. into wild-type C57BL/6 (CD45.1) mice either at the time of tumour cell injection or 7 days later. For some experiments, CTLs were incubated overnight with BTP2 (or DMSO) prior to injection to inhibit CRAC channels.

### Cytotoxicity and degranulation assays

CD8^+^ T cells from wild-type OT-1 or *Stim1*^*fl/fl*^*Stim2*^*fl/fl*^ *Cd4Cre OT-1* mice were differentiated into CTLs as described above. EG7-Ova T-cell lymphoma cells were labelled with 5 µM CellTracker Orange for 10 min at 37°C. 1 × 10^5^ CTLs were coincubated with 0.5–5 × 10^5^ EG7-Ova cells for 90 min at 37°C after which cells were stained with Annexin V–FITC or Annexin V–eFluor 450 and anti-CD8 antibody to measure apoptosis in CD8^−^ EG7-Ova tumour cells. To analyse lytic granule exocytosis, CTLs were stained with antibodies against CD8 and CD107a and analysed by flow cytometry.

### Statistical Analyses

*p* Values were calculated using the two-tailed unpaired Student's *t*-test and Graphpad Prism software. All graphs show the average ± SEM.

## For more information

Feske lab: http://labs.pathology.med.nyu.edu/feske-lab/

## Supplementary material

As a service to our authors and readers, this journal provides supporting information supplied by the authors. Such materials are peer reviewed and may be re-organized for online delivery, but are not copy-edited or typeset. Technical support issues arising from supporting information (other than missing files) should be addressed to the authors.
