# Supplementary material for: STIM1 and STIM2-mediated Ca2+ influx regulates antitumour immunity by CD8+ T cells
Source: EMBO Mol Med. 2013 Aug 6;5(9):1311–21. doi: 10.1002/emmm.201302989 (PMC3799488; doi:10.1002/emmm.201302989)
Supplement: Supplementary file 1 [file emmm0005-1311-SD1.pdf]

EMBO Molecular Medicine

STIM1 and STIM2-mediated Ca^{2+} influx regulates antitumor immunity by $CD8^{+}$ T cells

Carl Weidinger, Patrick Shaw and Stefan Feske

Corresponding author: Stefan Feske, NYU

Review timeline:

Submission date:	01 May 2013
Editorial Decision:	03 June 2013
Revision received:	11 June 2013
Editorial Decision:	28 June 2013
Accepted:	28 June 2013

Transaction Report:

(Note: With the exception of the correction of typographical or spelling errors that could be a source of ambiguity, letters and reports are not edited. The original formatting of letters and referee reports may not be reflected in this compilation.)

1st Editorial Decision 03 June 2013

Thank you for the submission of your manuscript to EMBO Molecular Medicine. We have now heard back from the three referees whom we asked to evaluate your manuscript.

As you will see from the enclosed reports, the referees are overall very enthusiastic about the study and only have a few concerns that I would like you to address experimentally when appropriate.

Should you be able to address these concerns in full, we would be willing to consider a revised manuscript and depending on the nature of the revisions, this may be sent back to the referees for another round of review. Please note that that it is our journal's policy to allow only a single round of revision, and that acceptance or rejection of the manuscript will therefore depend on the completeness of your response and the satisfaction of the referees with it.

I look forward to seeing a revised form of your manuscript as soon as possible.

***** Reviewer's comments *****

Referee #1 (Comments on Novelty/Model System):

The authors demonstrate very convincingly that STIM proteins are required for antitumour immune responses of CD8+ T cells. Experimental approach is very complete including carefully elaborated in vitro and in vivo experiments, which are neatly performed. Results are cristal-clear. The topic is novel and has a very high medical impact, as it is directly related to mechanisms for tumour progression. It is striking that failure of both, STIM1 and STIM2, is required for neat effect on degranulation, expression of FasL and cytokine production (whereas Orai1 KO is enough). This makes a lot of sense and may explain previous confusing results. While SOCE inhibitors may antagonize carcinogenesis, the present results caution possible therapeutic use, as they could facilitate engraftment by interfering with antitumour immunity thus favouring tumour progression.

Referee #1 (Remarks):

My overall opinion is that the paper is technically sound, novel and with high medical impact. Results are clear and their interpretations convincing and very clearly written. I do not think that it would be significantly improved by any changes. In summary, I consider that the paper is excellent and it should be published as it is.

Referee #2 (Remarks):

Overall, this is good paper with appropriate experiments and controls.

The authors show for the first time that SOCE mediated by STIM1 and STIM2 is required for anti-tumor immune responses. They make lovely use of conditional KO mice with T-cell specific deletion of stim1 & stim2 genes. Using these mice, they show that by mediating SOCE, STIM1 and STIM2 allow CD8+ T cells to prevent the engraftment of timor cells and to curtail tumor growth. Therefore, as the authors discuss, their data have important implications for the use of drugs targeting CRAC channels since inhibition of SOCE will compromise anti-tumor immunity mediated by CD8+ T cells.

The authors also investigate whether STIM proteins contribute to the cytotoxic function of CD8+ T cells. Fig. 4A shows that STIM1 and STIM2 deletion (use of DKO) impairs CD8+ T cell-mediated killing of tumor cells and also a reduction of IFNg and TNFa production (Fig. 4B). However, the authors do not show a specific requirement of STIM1/2 for degranulation (i.e. exocytosis of cytolytic granules). They do show that inhibition of CRAC channels using BTP2 (at a high (maximal) concentration of 1 uM) reduces cell surface expression of CD107a by ~50%. There is no discussion as to what could mediate this remaining 50% - eg. other sources of calcium-ER or acidic stores.
To satisfy their claim that STIM1 and STIM2 are required for degranulation, the authors need to show CD107a cell surface expression using the WT and DKO CD8+ T cells after coincubation with EG7-Ova cells. Additionally they should show that these cells contain similar amounts of Granzyme B and Perforin proteins (as in Fig. 5B-C, using BTP2 inhibitor of CRAC).

In the discussion, p. 9, paragraph 3, I'm a little confused. I think that are saying that only moderate levels of SOCE are sufficient for CTL function, i.e. cytotoxicity. There is no discussion for an alternative source of Ca2+, i.e the acidic Ca2+ store: the cytolytic granule.

A few minor points:
Fig 1B - is there any significant difference between % Treg cells in WT vs DKO?
Fig. 2A - "no apparent differences in cell numbers" - please count cells in a given area. Only representative images are shown.
Fig. 2E - why ratio BTP2/DMSO-treated CTL? Do DMSO-treated CTL home to TDLN?
p.7 - total numbers of cTL in spleens and TDLN WT vs DKO data not shown - please give % in the text.

Referee #3 (Comments on Novelty/Model System):

This is a very interesting paper that provides details of how SOCE controls CTL function.

Referee #3 (Remarks):

Cytotoxic Lymphocytes (CTL) have fundamental role in anti-tumor responses. In the manuscript by Weidinger et al., the role of store operated calcium entry (SOCE) is examined a critical signal transduction pathway controlling CTL responsiveness and effector function. The authors utilize CTL cells and mice lacking STIM1/2 (double KO mice (DKO)) or pharmacologic inhibition of Orai1 channels to show that CD8+ cells lacking SOCE are unable to control tumor engraftment and proliferation. These studies are very important and represent a significant advancement to

understanding of SOCE in immune function. I think a few questions should be answered before publication.
1. The in vivo work is really important and elegant. I wonder in tumors with more antigenicity would not require the addition of CD25 blockade.
2. What happens if the in vivo experiments with DKO are performed with BTP-2, is it additive?
3. Why is STIM2 required? This might imply STIM proteins are functioning independent of Orai1 channels.
4. Would STIM mutants enable the authors to separate the effects on gene expression and degranulation?

1st Revision - authors' response 11 June 2013

Referee #1

[...] My overall opinion is that the paper is technically sound, novel and with high medical impact. Results are clear and their interpretations convincing and very clearly written. I do not think that it would be significantly improved by any changes. In summary, I consider that the paper is excellent and it should be published as it is.

Response: We thank the reviewer for his/her generous comments.

Referee #2

Overall, this is a good paper with appropriate experiments and controls.

The authors show for the first time that SOCE mediated by STIM1 and STIM2 is required for anti-tumor immune responses. They make lovely use of conditional KO mice with T-cell specific deletion of stim1 & stim2 genes. Using these mice, they show that by mediating SOCE, STIM1 and STIM2 allow CD8+ T cells to prevent the engraftment of tumor cells and to curtail tumor growth. Therefore, as the authors discuss, their data have important implications for the use of drugs targeting CRAC channels since inhibition of SOCE will compromise anti-tumor immunity mediated by CD8+ T cells.

The authors also investigate whether STIM proteins contribute to the cytotoxic function of CD8+ T cells. Fig. 4A shows that STIM1 and STIM2 deletion (use of DKO) impairs CD8+ T cell-mediated killing of tumor cells and also a reduction of IFNg and TNFa production (Fig. 4B). However, the authors do not show a specific requirement of STIM1/2 for degranulation (i.e. exocytosis of cytolytic granules). They do show that inhibition of CRAC channels using BTP2 (at a high (maximal) concentration of 1 uM) reduces cell surface expression of CD107a by ~50%. There is no discussion as to what could mediate this remaining 50% - eg. other sources of calcium-ER or acidic stores.

To satisfy their claim that STIM1 and STIM2 are required for degranulation, the authors need to show CD107a cell surface expression using the WT and DKO CD8+ T cells after coincubation with EG7-Ova cells. Additionally they should show that these cells contain similar amounts of Granzyme B and Perforin proteins (as in Fig. 5B-C, using BTP2 inhibitor of CRAC).

Response: We thank the reviewer for his/her very positive comments. We completely agree that it would be interesting and important to investigate how the genetic deletion of *Stim1/Stim2* genes in $CD8^+$ T cells affects the degranulation of CTL and the expression of Granzyme B and Perforin. We have conducted preliminary experiments to analyze granzyme B expression in CTL from *Stim1*$^{fl/fl}$ *Stim2*$^{fl/fl}$ *Cd4Cre OT-1* mice after restimulation with either Ionomycin/PMA or Ova peptide. We

found that granzyme B levels were moderately reduced in STIM1/2-deficient CTL compared to wild-type CTL. (Expression levels of perforin were not determined in this experiment). (Figure A for reviewers).

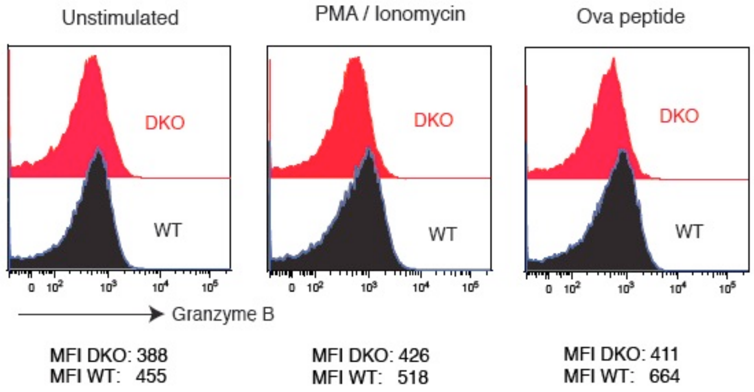

Figure A: SOCE is required to control tumor growth *in vivo.* Wildtype (WT) and *Stim1*$^{fl/fl}$*Stim2*$^{fl/fl}$ *Cd4Cre* (DKO) mice were injected with 1x10^{5} EG7 cells, which are derived from the murine T cell lymphoma EL4 and stably express ovalbumin (Ova). Recipient mice were monitored for tumor size over 18 days. Three mice per group.

The following sentence has been added to the results section on page 8: "A moderate defect in granzyme B protein expression was observed in CTLs from *Stim1*$^{fl/fl}$ *Stim2*$^{fl/fl}$ *Cd4Cre OT-1* mice (that lack all SOCE) following strong stimulation with PMA/ionomycin or Ova peptide (unpublished observations)". These results were not included in the original submission because the experiment was done only once. Unfortunately, we were unable to repeat these experiments because we lost almost our entire colony of conditional knock-out mice including *Stim1*$^{fl/fl}$*Stim2*$^{fl/fl}$ *Cd4Cre* and *Stim1*$^{fl/fl}$*Stim2*$^{fl/fl}$ *Cd4Cre OT-1* mice during hurricane Sandy, which hit New York City and NYU Medical Center on 29th October 2012 and flooded the vivarium in which our mouse strains were housed:

http://www.nytimes.com/2012/11/10/nyregion/damage-from-hurricane-sandy-could-cost-nyu-langone-millions.html?_r=0

http://www.nature.com/news/researchers-battle-storm-s-wrath-1.11756

We are currently in the process of rederiving the mouse strains used in this study. We estimate that *Stim1*$^{fl/fl}$*Stim2*$^{fl/fl}$ *Cd4Cre* mice will be available for future experiments by the end of September 2013 and *Stim1*$^{fl/fl}$*Stim2*$^{fl/fl}$ *Cd4Cre OT1* mice after additional crossing at the end of 2013. While the experiments requested by the reviewer themselves are not difficult to conduct, the combined time for rederivation of mouse strains and performance of these experiments would take ~ 6-8 months. This was the reason why we have analyzed (i) CD107a expression/degranulation and (ii) perforin and granzyme B levels in cells treated with BTP2, which – as shown in Figure 4C – suppresses Ca^{2+} influx almost completely and to an extent similar to that in STIM1/STIM2 deficient $CD8^{+}$ T cells. We hope that the editors and reviewer understand our currently limited options to respond to their requests.

There is no discussion as to what could mediate this remaining 50% - eg. other sources of calcium- ER or acidic stores.

Response: It is possible that Ca^{2+} release from intracellular stores (ER, acidic stores) is sufficient to mediate residual (50% of maximum) degranulation in STIM1/2-deficient CTL because ER Ca^{2+} stores are filled in these cells. Alternatively, degranulation may be only partially Ca^{2+} dependent. Our response to reviewer #3 includes a more detailed discussion of the potential mechanisms by

which Ca^{2+} regulates lytic granule exocytosis – please see further below. It is important to emphasize that the exact Ca^{2+} requirements of this process are not known.

In the discussion, p. 9, paragraph 3, I'm a little confused. I think that are saying that only moderate levels of SOCE are sufficient for CTL function, i.e. cytotoxicity. There is no discussion for an alternative source of Ca2+, i.e the acidic Ca2+ store: the cytolytic granule.

Response: We agree with the summary of the reviewer "that only moderate levels of SOCE are sufficient for CTL function, i.e. cytotoxicity". However, there is no need to postulate other sources of Ca^{2+}, either intracellular Ca^{2+} stores or Ca^{2+} influx channels in the plasma membrane. To make our interpretation more clear, we are showing Figure B for the reviewers' evaluation and have added the following sentences to the discussion on p.9, paragraph 3: "Consistent with these findings, we observed normal rejection of tumor cells in Treg-depleted *Stim1^fl/fl^ Cd4Cre* mice *in vivo* compared to WT littermates (unpublished observations). STIM1 and STIM2 have synergistic roles in CRAC channel activation and both molecules are required for full SOCE in $CD8^+$ T cells. STIM1 and STIM2 act with different kinetics and thresholds of activation in response to store depletion (Shaw et al, 2012). Only the combined deletion of *Stim1* and *Stim2* genes completely abolishes SOCE. Deletion of *Stim1* alone strongly, but incompletely reduces SOCE. By contrast, deletion of *Stim2* alone has no effect on the peak of Ca^{2+} influx but significantly impairs sustained SOCE (Oh-Hora et al, 2008). Normal tumor rejection in *Stim1^fl/fl^ Cd4Cre* mice therefore indicates that moderate SOCE is sufficient for the cytolytic effector functions of CTLs and their ability to provide antitumor immunity *in vivo*". Consistent with these findings, $CD8^+$ T cells from a human patient with a loss-of-function mutation in the *STIM1* gene showed normal cytolytic function of tumor cells *in vitro* (Fuchs et al. *J Immunol.* 2012, 188:1523-33).

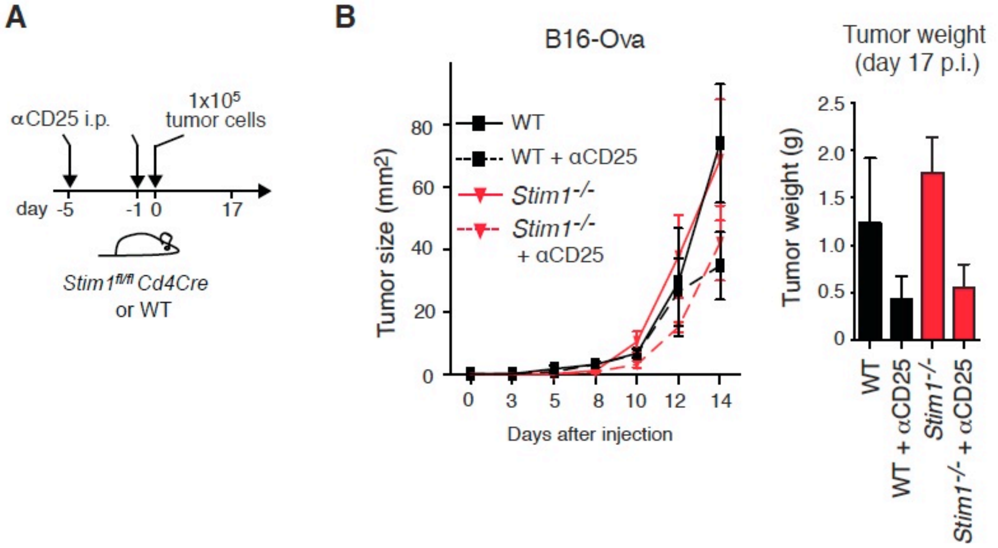

Figure B: T cell specific deletion of STIM1 alone does not impair antitumor immunity. (A) Allogenic tumor transfer to Treg cell-depleted mice. Wildtype (WT) and *Stim1^fl/fl^ Cd4Cre* mice were injected i.p. with 250 µg anti-CD25 antibody (PC61) to deplete Treg cells (or 1x PBS as control) on days -5 and day -1 before intradermal (i.d.) tumor inocculation with $1x10^5$ B16-Ova melanoma cells. (B) Tumor growth in WT and *Stim1^fl/fl^ Cd4Cre* (*Stim1^-/-^*) mice. WT (black) and *Stim1^-/-^* (red) mice injected with B16-Ova melanoma were monitored for tumor size (left panel). 17 days later, mice were sacrificed and tumor weight was measured. Dashed lines represent mice injected with anti-CD25 antibody. Three mice per group.

A few minor points:

Fig 1B - is there any significant difference between % Treg cells in WT vs DKO?

© EMBO

Response: There is a significant differences in the percentage of Treg cells in WT vs DKO mice. The p value has been added to Figure 1B. These results are in line with previous results from *Oh-hora et al. Nat Immunol. 2008 9:432-43*.

Fig. 2A - "no apparent differences in cell numbers" - please count cells in a given area. Only representative images are shown.

Response: We thank the reviewer for this suggestion. We have included an analysis of the number of T cells in the tumors in the revised Figure 2B.

Fig. 2E - why ratio BTP2/DMSO-treated CTL? Do DMSO-treated CTL home to TDLN?

Response: We apologize for not explaining the experimental setup well enough. We coinjected SOCE-depleted (BTP2-treated) or SOCE-sufficient (DMSO treated) CTL at a 1:1 ratio and compared their ratio before injection and 60 min after injection (CTL retrieved from TDLN). BTP2-treated CTL homed to TDLN to the same extent as DMSO-treated CTL, indicating that SOCE is not required for CTL migration. We have rewritten the description of the experiment in the results section.

p.7 - total numbers of CTL in spleens and TDLN WT vs DKO data not shown - please give % in the text.

Response: We have included these numbers in the results text referring to Figure 3 on page 7.

Referee #3

Cytotoxic Lymphocytes (CTL) have fundamental role in anti-tumor responses. In the manuscript by Weidinger et al., the role of store operated calcium entry (SOCE) is examined a critical signal transduction pathway controlling CTL responsiveness and effector function. The authors utilize CTL cells and mice lacking STIM1/2 (double KO mice (DKO)) or pharmacologic inhibition of Orai1 channels to show that CD8+ cells lacking SOCE are unable to control tumor engraftment and proliferation. These studies are very important and represent a significant advancement to understanding of SOCE in immune function. I think a few questions should be answered before publication.

1. The in vivo work is really important and elegant. I wonder in tumors with more antigenicity would not require the addition of CD25 blockade.

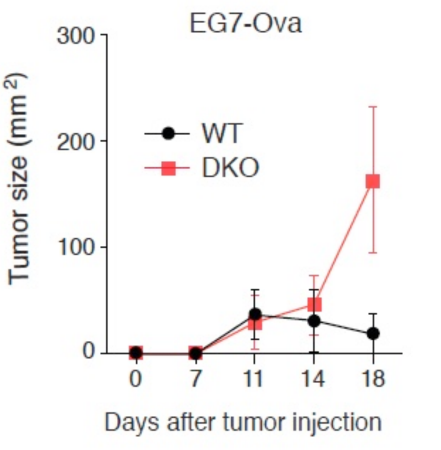

Figure C: SOCE is required to control tumor growth *in vivo*. Wildtype (WT) and *Stim1*$^{fl/fl}$*Stim2*$^{fl/fl}$ *Cd4Cre* (DKO) mice were injected with 1x10^5 EG7 cells, which are derived from the murine T cell lymphoma EL4 and stably express ovalbumin (Ova). Recipient mice were monitored for tumor size over 18 days. Three mice per group.

Response: This is an interesting point and we agree that in a more immunogenic tumor model, the deletion of Treg cells may not be needed to observe defects in antitumor immunity by SOCE-deficient CD8 T cells. Indeed, we have conducted experiments transferring syngeneic EG7 tumor cells to WT and *Stim1*$^{fl/fl}$ *Stim2*$^{fl/fl}$ *Cd4Cre* mice without prior deletion of Treg cells. EG7 cells are derived from the murine T-cell lymphoma EL-4 and stably express ovalbumin (OVA). Compared to B16 and MC38 tumor cells, EG7 cells are more immunogenic allowing CD8$^+$ T cells to spontaneously reject tumors (Sondergaard H et al., *J Immunol* 2009; 183:7326-7336). Reported rejection rates in the literature are very variable even within the same experiment (on average ~ 50%), making the results somewhat difficult to interpret. In

preliminary experiments, we observed that WT mice successfully rejected EG7 tumor cells despite initial tumor growth, whereas tumors continuously grew in *Stim1$^{fl/fl}$ Stim2$^{fl/fl}$ Cd4Cre* mice, indicating that SOCE mediated by STIM1 and STIM2 is required for the rejection of EG7 tumors (see Figure C for the reviewers' evaluation). We added the following sentence to the results section on page 5: "Similar observations were made without prior depletion of Treg cells when WT and DKO mice were injected with more immunogenic EG7 lymphoma cells. Under these conditions, WT mice successfully rejected EG7 tumor cells despite initial tumor growth, whereas tumors continuously grew in *Stim1$^{fl/fl}$ Stim2$^{fl/fl}$ Cd4Cre* mice (unpublished observations)". The number of mice analyzed per group is low (n=3) and we are currently not able to repeat the experiment due the unavailability of *Stim1$^{fl/fl}$ Stim2$^{fl/fl}$ Cd4Cre* mice (as discussed above). We had therefore not included these data in the original version of the manuscript.

2. What happens if the in vivo experiments with DKO are performed with BTP-2, is it additive?

Response: BTP2 is considered to be a selective CRAC channel inhibitor (*Zitt C et al., J Biol Chem 2004 279:12427-37*). STIM1 and STIM2 are essential for CRAC channel activation and deletion of both *Stim1* and *Stim2* genes completely abolishes CRAC channel function and SOCE (*Oh-hora et al. Nat Immunol. 2008 9:432-43*). We therefore do not expect that BTP2 has additional inhibitory effects on Ca^{2+} influx as it targets the same pathway.

3. Why is STIM2 required? This might imply STIM proteins are functioning independent of Orai1 channels.

Response: To directly answer the referee's question, there is no need to postulate that STIM proteins function independent of ORAI1 to explain our data.

The response to this question is similar to that given to referee #1 above: "Both STIM1 and STIM2 are required to fully activate CRAC channels in $CD8^+$ T cells as only combined deletion of *Stim1* and *Stim2* gene expression completely abolishes SOCE. Deletion of *Stim1* alone strongly, but incompletely reduces SOCE, whereas deletion of *Stim2* alone has no effect on the peak of Ca^{2+} influx but significantly impairs sustained SOCE (*Oh-hora et al. Nat Immunol. 2008 9:432-43*)". Therefore STIM1 and STIM2 have distinct but synergistic roles in CRAC channel activation. Both molecules are able to activate CRAC channels and cause SOCE; however, they do so with different kinetics and thresholds of activation.

Briefly, upon depletion of Ca2+ from ER stores, Ca^{2+} dissociates from an EF-hand domain in the N ER-lumenal N terminus of STIM1 and STIM2, which results in a conformational change in the N termini and the activation of STIM molecules. The kinetics with which the NT of STIM1 unfolds was reported to be > 3-fold faster when compared to STIM2. This difference resulted in a much faster (by ~ 70-fold) conversion of STIM1 from a monomeric to an oligomeric (i.e. activate) state compared to STIM2. STIM1 and STIM2 also differ significantly with regard to their activation thresholds, i.e. the decrease in $[Ca^{2+}]_{ER}$ that leads to their activation. Half-maximal STIM1 activation is achieved when $[Ca^{2+}]_{ER}$ falls below ~ 400 mM, whereas STIM2 responds to small decreases in $[Ca^{2+}]_{ER}$ (from the estimated resting concentration of ~ 800 mM). It has therefore been suggested that STIM2 activates SOCE upon small fluctuations in $[Ca^{2+}]_{ER}$ and thus maintains the filling of ER stores and basal $[Ca^{2+}]_i$ in resting cells. By contrast, STIM1 is responsible for SOCE in response to cellular stimulation through, for instance, antigen receptors on lymphocytes. Furthermore, expression levels of STIM1 in lymphocytes are typically much higher than those of STIM2. Together, these differences in the functional properties and expression levels of STIM1 and STIM2 account for their quantitatively different contributions to SOCE in T cells. (A slightly more detailed summary of the distinct but synergistic roles of STIM1 and STIM2 in CRAC channel activation can be found in a review we recently published: *Shaw PJ et al. Cell Mol Life Sci 2012 Oct 5, Epub ahead of print*).

4. Would STIM mutants enable the authors to separate the effects on gene expression and degranulation?

Response: We are not quite sure what type of mutations the reviewer is thinking of. A direct association of STIM1 or STIM2 molecules with the lytic granule exocytosis machinery that could be modulated by mutations, as suggested by the reviewer, has not been reported and seems rather unlikely. STIM1 and STIM2 activate CRAC channels and Ca^{2+} influx, which may regulate degranulation either by posttranslational effects on the cytolytic granule exocytosis machinery or by controlling the expression of proteins that are part of this machinery. The polarization of the microtubule organizing center (MTOC), required for targeted release of cytolytic granules was shown to be independent of Ca^{2+}, whereas granule polarization was mediated by Ca^{2+} signalling (*Quann et al. Nature Immunol. 2009, 10:627–635*). At the neurological synapse – an often used model for the lytic immune synapse between CTL and target cells – Ca^{2+} binds to the Ca^{2+} sensor synaptotagmin 1 on the synaptic vesicle membrane and allows it to associate with the membrane docking complex, thereby localizing vesicles close to Ca^{2+} channels required for triggering the last step of vesicle exocytosis. In CTLs, synaptotagmin 7 has been proposed as a candidate Ca^{2+} sensor of cytotoxic granule exocytosis (reviewed in *de Saint Basile et al. Nature Rev Imm 2010, 11:568-79*). It is possible that SOCE mediated by CRAC channels provides the Ca^{2+} signal required for the fusion of cytolytic granules with the plasma membrane, but this has not been shown directly yet.

2nd Editorial Decision 28 June 2013

Please find enclosed the final report on your manuscript. We are pleased to inform you that your article is accepted for publication and is now being sent to our publisher to be included in the next available issue of EMBO Molecular Medicine.

Congratulations on your interesting work.
